# The Application and Value of 3T Magnetic Resonance Imaging in the Display of Pulmonary Nodules

**DOI:** 10.3389/fonc.2022.844514

**Published:** 2022-05-18

**Authors:** Hui Feng, Gaofeng Shi, Hui Liu, Qian Xu, Lijia Wang, Ning Zhang

**Affiliations:** Department of Radiology, The Fourth Hospital of Hebei Medical University, Shijiazhuang, China

**Keywords:** magnetic resonance imaging, pulmonary nodules, multi-sequence, display ability, CT

## Abstract

**Objective:**

The aim of this study was to evaluate the sensitivity and accuracy of multi-sequence 3T magnetic resonance imaging (MRI) in the detection of different types of pulmonary nodules.

**Methods:**

A total of 68 patients with pulmonary nodules identified using computed tomography (CT) subsequently underwent MRI. Using CT images with a slice thickness of 1 mm as the gold standard, the sensitivity of three MRI sequences in detecting different types of pulmonary nodules was calculated, and the image quality was also evaluated. Nodule types included solid nodules, ground glass nodules (GGN), and part-solid nodules (PSN). Statistical analyses of data were conducted using the software SPSS 21.0. The intra-class correlation coefficient was calculated in order to compare the consistency of nodule size in both MRI and CT.

**Results:**

CT detected 188 pulmonary nodules in 68 patients, including 87 solid nodules and 101 sub-solid nodules, the latter comprising 46 PSNs and 55 GGNs. The average nodule diameter was approximately 7.7 mm. The sensitivity of MRI in detecting nodules ≥ 6 mm in diameter and those of > 8 mm in diameter was 92% and 100%, respectively, and the sequence with the highest detection rate was T2-BLADE. In relation to solid nodules, the sequence with the highest detection rate was T1 Star-VIBE, while the T2-BLADE sequence demonstrated the highest detection rate of sub-solid nodules. The image quality of the T1 Star-VIBE sequence was better than that of both the T2-HASTE and the T2-BLADE sequences. The consistency of CT and MRI sequences for nodule size was high with a consistency coefficient of 0.94–0.98.

**Conclusion:**

The detection rate of MRI for nodules with a diameter of > 8 mm was 100%. The T2-BLADE sequence had the highest detection sensitivity. The sequence with the best image quality was the T1 Star-VIBE.

## Introduction

At present, lung cancer is one of the main causes of cancer-related death worldwide, and the 5-year survival rate is < 14% (1). Early detection, early diagnosis, and early treatment are crucial in improving the survival rate of lung cancer patients (2). With the worldwide development of early lung cancer screening, and the wide application of low-dose computed tomography (LDCT), the mortality of lung cancer has been reduced (3–5). Early lung cancer chiefly manifests as intrapulmonary solitary pulmonary nodules (6). The term solitary pulmonary nodule refers to a single, round or quasi-round nodule in the lung with a clear boundary, a diameter of ≤ 3 cm, without atelectasis, pulmonary lymphadenopathy, or pleural effusion (7). Pulmonary nodules are divided into three types according to their density: solid nodules, part-solid nodules (PSNs), and ground glass nodules (GGNs). CT is the most commonly used method to detect and evaluate pulmonary nodules. In comparison to chest radiography, the radiation dose of CT is higher, and CT may increase the risk of radiation-induced cancer, especially in those patients requiring multiple follow-up imaging. It is especially in pediatric population due to they are more sensitive to radiation damage than adults and their longer life span (8). It has been estimated that when LDCT screening is performed between the ages of 50 and 75, the proportion of radiation-induced malignant tumors accounts for approximately 0.5%–5.5% of all the screened population (9–11). Therefore, in consideration of the issue of radiation safety, magnetic resonance imaging (MRI) is now used more and more in the evaluation of chest diseases. Recently, MRI in general, and especially 3T high-field MRI, has become a research hotspot.

In recent years, MRI technology has developed rapidly: high-performance gradient systems, parallel acquisition, improved magnetic field uniformity, and the use of multi-channel coils have all improved the temporal and spatial resolution, so that MRI can be used for lung imaging (10–14). Furthermore, as MRI has good soft tissue resolution and no ionizing radiation, it can be used as a good supplement or alternative method to CT diagnosis (15–19).

At present, many MRI sequences exist for the detection and display of small pulmonary nodules, although they are still not widely applied in clinical practice. A number of studies comparing chest MRI with CT have found that there are great differences in the diagnostic efficacy in the detection of pulmonary nodules with a diameter of < 5 mm, with an accuracy of 35%–84% (20–24). The purpose of this study was to investigate the ability of conventional MRI sequences such as T2-HASTE, T1-Star-VIBE, and T2-BLADE to detect small pulmonary nodules.

## Materials and Methods

### Subjects

Patients who underwent chest CT examination in our hospital from May 2017 to December 2019 and were found to have pulmonary nodules were enrolled into this study. This prospective study was approved by the hospital ethics committee. All patients provided written informed consent before examination.

Inclusion Criteria: patients who were found to have pulmonary nodules with a diameter of 6–30 mm on chest CT, including solid nodules, PSNs, and GGNs, and agreed and accepted to undergo MRI imaging.

Exclusion Criteria: (1) patients with calcified nodules, (2) patients with contraindications to MRI, including cardiac pacemakers, metal implants, and claustrophobia, and (3) patients whose images could not be evaluated due to severe artifacts.

The interval between CT and MRI scanning did not exceed 24 hours.

### Scan parameters

#### CT Scanning Parameters

CT was conducted using a Siemens second-generation dual-source CT machine (Definition Flash, Siemens Healthcare, Forchheim, Germany). The following parameters were used: tube voltage 120 KV, tube current auto-milliampere second, field of view 300 mm, slice thickness 1 mm, matrix 512 × 512, 1.2 of pitch, reconstruction interval 1 mm, and convolution kernel B70f. The patient lay on his/her back on the examination table with his/her hands raised, and scanning was performed at the end of inspiration after breath holding. The scanning range was from the thoracic entrance to the level of the diaphragm and covered the entire lung. The scanning time was 3.98 seconds, and the effective dose was 1.2–1.5 mSv.

#### MRI Scanning Parameters

MRI was performed using a Siemens 3T MRI scanner (Magnetom Skyra; Siemens Healthcare), and a multi-channel phased array body coil was used. In order to reduce the heartbeat and respiratory movement artifacts, respiratory gating was used, and the patient was supine with his/her hands raised. Scan sequences: T2-BLADE, T1 Star-VIBE, and T2-HASTE. Scanning parameters are presented in **Table 1**.

**Table 1:** Parameters of the applied MRI sequences.

Parameters	T2-BLADE	T2-HASTE	T1-star VIBE
TR (ms)	4000	1600	6.65
TE/TEs (ms)	79	96	3.17
Plane	Axial	Axial	Axial
FOV (mm)	380	380	420
RecFOV (%)	100	75	100
Matrix	320*275	320*224	320*192
Slice thickness (mm)	3	4	3
Breath-hold	NO	YES	NO

### Image Analysis

#### Analysis of the CT Images

CT images were analyzed by a radiologist with seven years of working experience. The CT images were observed in the thin-layer image of lung window, and the window width and the window level were fixed at 1200 HU and − 600 HU, respectively. The location of the nodules, the maximum axial diameter (mm), the average diameter (mm), and the CT value (HU) were recorded.

#### Analysis of the MRI Images

MRI image analysis was independently completed by two radiologists with 5 and 10 years of working experience, respectively, who were blinded to the CT results. For the MRI images, whether the nodules were displayed, the location, and axial mean diameter of the nodules (mm) were recorded.

#### Image Quality Evaluation

The image quality was evaluated subjectively and objectively by two radiologists with 6 and 10 years of radiology experience.

Subjective evaluation was mainly based on image quality, lesion display, and artifacts. A 5-point system was adopted to evaluate the image quality of each scanning sequence using the following evaluation criteria. Score=1: the image is blurred with obvious artifacts and cannot be used for diagnosis, Score=2: the image is blurred, there are obvious artifacts on some layers, but it does not affect the diagnosis, Score=3: there is an indistinct tissue boundary with slight artifact on some layers, but the image can be used for diagnosis, Score=4: the tissue structure boundary is slightly blurred and there are minor artifacts, but the image can be used for diagnosis, and Score=5: the tissue structure boundary is clear without artifacts, and the image can be used for diagnosis (25, 26).

Objective evaluation included calculating the signal-to-noise ratio (SNR) and the contrast-to-noise ratio (CNR) of the images (27). The signal intensities of the lung parenchyma in the anterior and posterior regions of the lung were measured in order to obtain the average signal intensity value of the lung parenchyma (SI_lung_), while attempting to avoid the measurement from covering pulmonary vessels and keeping at least 2 cm away from pleura. Signal intensities of muscle (SI_muscle)_ and background (SI_background)_ were simultaneously measured. The SI_background_ is the average value of the region of interest in the front, back, left, and right regions of the background.

The SNR = SI_lung_/SD, and the CNR = (SI_muscle_ - SI_lung_)/SD, where the SD is the average of the background standard deviation.

#### Comparison of CT and Various MRI Sequences in Detecting Nodules

CT was regarded as the gold standard for the detection of nodules. The number of nodules detected in different locations, the sizes and types of nodules, as well as the sensitivity of each MRI sequence were calculated. According to the MRI findings, the display results were divided into the following categories: lesions being completely displayed, lesions being partially displayed, and lesions not being displayed. The categories of completely displayed and partially displayed nodules were considered to be detectable. False positivity was defined as nodules that were not detected by CT but were considered positive on MRI.

### Statistical Analysis

Statistical analysis was performed using the SPSS 21.0 software (IBM, Armonk, NY, US). Taking the CT result as the gold standard for nodule detection, the sensitivity of each MRI sequence in detecting nodules with different locations, sizes (Fleischer Association nodule classification), and of different types was calculated. Measurement data were compared using the rank sum test, count data were compared using the X^2^ test, and categorical variables were compared using McNemar’s test. The Bland–Altman diagram was used to analyze and compare the size difference of pulmonary nodules detected by CT and MRI. The Kappa test was used to compare the consistency of image quality, and the intra-class correlation coefficient was used to evaluate intra-observer and inter-observer consistency. A value of *P* < 0.05 was considered to be statistically significant.

## Results

### Patient Characteristics

A total of 77 patients underwent both CT and MRI: 37 were male and 40 were female. The age range was 47–75 years with an average age of 59 years. Seven cases of severe respiratory artifacts and two cases of claustrophobia were excluded. Consequently, a total of 68 patients, 33 male and 35 female, with an age range of 47–70 years and an average age of 56 years were included in the analysis.

### Results of the CT Image Analysis

A total of 188 pulmonary nodules were detected, including 87 solid nodules, 46 PSNs, and 55 GGNs. Among the solid nodules, 40 were < 6 mm in diameter, 15 nodules were 6–8 mm in diameter, and 32 nodules were > 8 mm in diameter. Among the PSNs, 19 nodules were < 6 mm in diameter and 27 nodules were > 6 mm in diameter. Among the GGNs, 30 nodules were < 6 mm in diameter and 25 nodules were > 6 mm in diameter. The average number of nodules was three per person, ranging from one to eight. Patient characteristics are presented in **Table 2**.

**Table 2:** Patient characteristics.

Characteristics	Finding
Age (y)	55.7 ± 10.3 (47-70)
Male (n=33)	56.8 ± 10.1 (47-68)
Female (n=35)	55.3 ± 9.90 (51-70)
**Nodule location**	
Middle and lower lobe	92
Upper lobe	96
**Nodule type**	
SN	87
PSN	46
GGN	55
**Diameter (mm)**	
Overall	7.7 ± 4.2 (3.4-21.1)
SN	7.2 ± 3.1 (3.4-20.3)
PSN	11.8 ± 5.4 (6.2-21.1)
GGN	6.9 ± 2.6 (4-17.7)

Data were presented as mean ± standard deviation. SN, solid nodule; PSN, partial solid nodule; GGN, ground glass nodule.

### Results of the MRI Image Analysis

#### Comparison of Nodule Detection Rate Between MRI Sequences

The sensitivity of detection of pulmonary nodules is presented in **Table 2**. A total of 188 nodules were detected from the CT images of 68 patients, with a diameter range of 3.4–21.1 mm. Forty patients had more than one lesion. A total of 144 nodules were detected by MRI with a detection rate of 76.5%. The individual detection rates for the 67 solid nodules, the 37 PSNs, and the 40 GGNs were 77%, 80.4%, and 72.7%, respectively. The detection rate of MRI for nodules with a diameter of > 8 mm was 95%.

**Figure 1:**
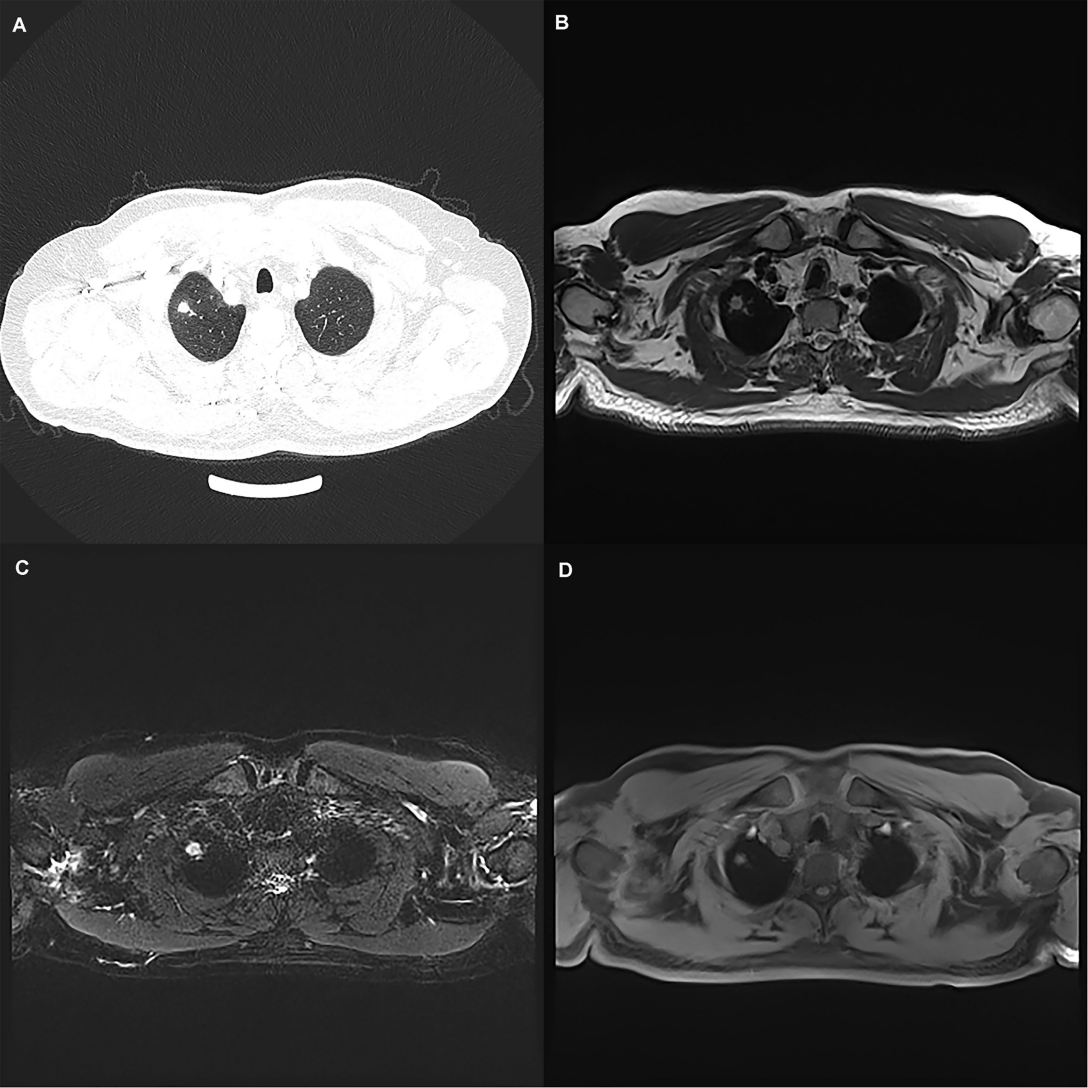
A 50-year-old man with a 7 mm pulmonary solid nodule (arrow) in the right upper lobe. The nodule is clearly seen on the computed tomography image **(A)** and the images from magnetic resonance image sequences T2-BLADE **(B)**, T2-HASTE **(C)**, and T1-Star-VIBE **(D)**. The arrows represent the location of the nodule.

**Figure 2:**
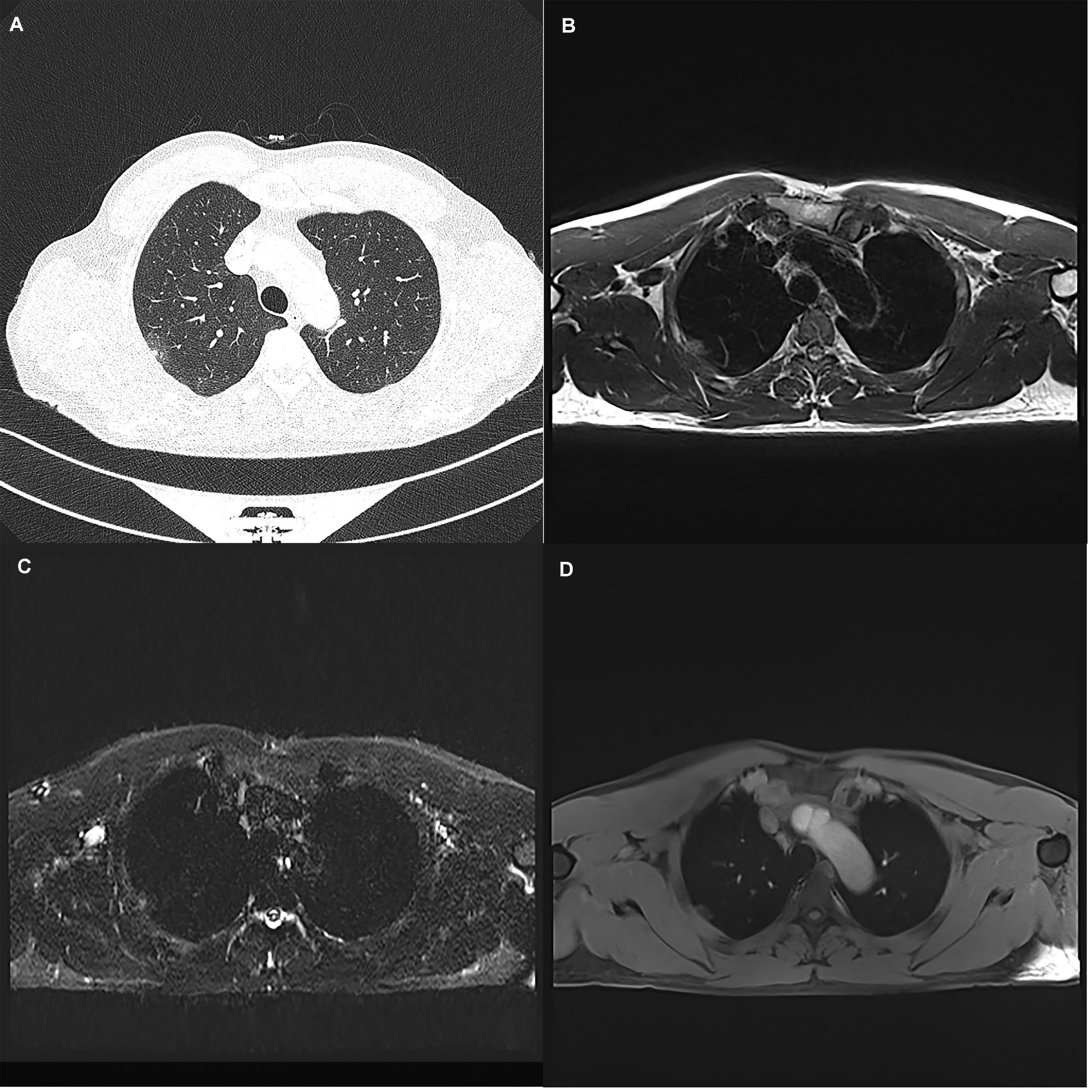
A 45-year-old woman with a 19 mm GGN (arrow) in the right upper lobe. The nodule is clearly seen on the computed tomography image **(A)** and the magnetic resonance image sequences T2-BLADE **(B)**, T2-HASTE **(C)**, and T1-Star-VIBE **(D)**. The arrows represent the location of the nodule.

For different types of nodules, the detection rate of MRI for solid nodules (77%) and PSNs (80.4%) was higher than that of GGNs (72.7%) (**Figures 1** and **2**). For the detection of nodules in different locations, the detection rate of nodules in the upper lobe was higher than that in the middle and lower lobes. The undetected nodules were mainly located in the mediastinum, the diaphragm, or around the heart.

The size range of nodules detected by MRI was 4–21.1 mm, with an average diameter of 9.8 mm. The maximum diameter of undetected nodules was 8 mm in diameter. The total number of undetected nodules was 44, including 40 nodules < 6 mm and 4 nodules ≥ 6 mm. Among the undetected nodules, solid nodules accounted for 50% (20/40), PSNs accounted for 22.5% (9/40), and GGNs accounted for 37.5% (15/40).

Comparing the nodule detection rates of the three MRI sequences, the T2-BLADE sequence demonstrated the highest sensitivity to detect nodules, followed by T2-HASTE and T1-Star-VIBE: (1) the detection rate of the T2-BLADE sequence was 71.3% with a total of 134 pulmonary nodules detected, including 62 solid nodules, 36 PSNs, and 37 GGNs, (2) the detection rate of the T2-HASTE sequence was 65.4% with a total of 123 pulmonary nodules detected, including 55 solid nodules, 35 PSNs, and 32 GGNs, and (3) the detection rate of the T1-Star-VIBE sequence was 54.8% with a total of 103 pulmonary nodules detected, including 66 solid nodules, 28 PSNs, and 9 GGNs.

The detection rates of nodules of different densities using different MRI sequences were also compared. In the case of solid nodules, the detection rates of the T1-Star-VIBE, T2-BLADE, and T2-HASTE sequences were 75.9%, 62%, and 63.2%, respectively. In the case of PSNs, the detection rates of the T1-Star-VIBE, T2-BLADE, and T2-HASTE sequences were 60.9%, 78.2%, and 76.1%, respectively. In the case of GGNs, the detection rates of the T1-Star-VIBE, T2-BLADE, and T2-HASTE sequences were 16.4%, 67.2%, and 58.1%, respectively. The detection rate of sub-solid nodules was higher than that of the GGNs with the T2-BLADE sequences yielding the highest detection rate of these nodules, followed by the T2-HASTE and T1-Star-VIBE sequences (**Table 3**).

**Table 3:** The sensitivity of the detection of different subgroups of pulmonary nodules.

Diameter of pulmonary nodules	Number of nodules	Detected by T1 star-VIBE	Detected by T2- BL- ADE	Detected by T2-HASTE
Solid nodule	87	66 (75.9%)	62 (71.3%)	55 (63.2%)
<6mm	40	20 (50%)	24 (60%)	18 (45%)
6-8mm	15	12 (80%)	13 (87%)	10 (66.7%)
>8mm	32	30 (93.7%)	29 (90.6%)	27 (84.3%)
Subsolid nodule	101	37 (36.7%)	72 (71.2%)	68 (67.3%)
PSN	46	28 (60.9%)	36 (78.2%)	35 (76.1%)
<6mm	19	9 (47.3%)	13 (68.4%)	11 (57.9%)
≥6mm	27	19 (70.3%)	23 (85.1%)	24 (88.9%)
GGN	55	9 (16.4%)	37 (67.2%)	32 (58.1%)
<6mm	30	4 (13.3%)	15 (50%)	12 (40%)
≥6mm	25	5 (20%)	22 (88%)	20 (80%)
Total	188	103 (54.8%)	134 (71.3%)	123 (65.4%)

The sensitivity was determined according to the Fleischner Society nodule size category.

#### Consistency Assessment of Nodule Size

The consistency of CT and MRI sequences in determining nodule size was high, and the range of consistency coefficient was 0.94–0.98 (**Table 4**).

**Table 4:** Mean diameter and mean difference of detected nodules for each sequence and correlation of MRI measurements compared to CT.

Sequence	Mean diameter of detected nodule by MRI (mm)	Mean difference of detected nodule by MRI compared to CT (mm)	ICC
T2-BLADE	6.52 ± 2.70	0.95 ± 0.34	0.98
T2-HASTE	6.69 ± 2.28	0.98 ± 0.39	0.94
T1-VIBE	6.09 ± 3.05	0.83 ± 0.35	0.98

Data were presented as mean ± standard deviation. ICC, inter-class correlation coefficient.

##### Image Quality Comparison

###### Subjective Evaluation

The consistency between the scores of the two observers was high, and the Kappa value was 0.895. Of the three sequences, the T1-Star-VIBE yielded more than 3 points (observer 1) in 66 cases, with T2-BLADE and T2-HASTE sequences yielding 3 points in 60 and 58 cases, respectively (**Table 5**).

**Table 5:** The result of image quality score.

Score	T1 star-VIBE		T2 BLADE		T2 HASTE	
	Observer 1	Observer 2	Observer 1	Observer 2	Observer 1	Observer 2
1	2	3	3	4	5	7
2	1	0	5	7	7	4
3	17	19	23	25	24	27
4	28	30	22	20	22	17
5	21	26	15	12	10	13

###### Objective Evaluation

The CNR and SNR of the T1-Star-VIBE sequence were the highest, followed by the T2-BLADE and T2-HASTE sequences (**Table 6**).

**Table 6:** Comparison of CNR and SNR in MRI sequences.

	T1 star-VIBE	T2-BLADE	T2-HASTE
CNR	44.45 ± 28.05	17.61 ± 16.364	9.92 ± 6.32
SNR	11.50 ± 4.96	4.23 ± 2.64	3.32 ± 1.75

Data were presented as mean ± standard deviation.

## Discussion

With the introduction of LDCT, the detection rate of pulmonary nodules has increased, although the majority of nodules detected are benign. While the likelihood of a malignant tumor originating from a small nodule is very low, as the size of the nodule increases, so does the probability of malignancy. Therefore, it is very important to be able to accurately determine the nodule size and monitor any changes. However, the accumulating radiation dose as a result of multiple CT scans will increase the risk of cancer (28–30). Many studies have proposed that MRI can be used as an investigation method in lung cancer screening (4, 31–34): the detection rate of pulmonary nodules by MRI largely depends on the size and also the nodule density (signal intensity). In addition, previous studies have highlighted the utility of short duration lung MRI in pediatric population also as a radiation free modality for the detection of pulmonary nodules (35, 36).

In this study, the sensitivity of MRI for detecting nodules ≥ 6 mm in diameter and > 8 mm in diameter was 92% and 100%, respectively, and these values are similar to results from recent research (33, 34). However, according to the Lung-RADS study, the malignant risk of nodules ≥ 6 mm in diameter was slightly higher than 1%, and short-term follow-up or further examination was required. The T2-BLADE sequence had the highest detection sensitivity. Regarding detection rate of different type of nodules, the T1 star-VIBE sequence had the highest detection rate for solid nodules and the T2-BLADE sequence had the highest detection rate for GGNs and PSNs.

In comparisons of the image quality among the three sequences, the sequence with the highest CNR and SNR was the T1-Star-VIBE, followed by the T2-BLADE and T2-HASTE sequences. The T1-Star-VIBE sequence adopts a radial manner in order to fill the K space in the image acquisition process resulting in less artifacts and improvement of the SNR of the image. This sequence is not sensitive to respiratory movement and can realize acquisition under free breathing. The T1-Star-VIBE sequence was therefore highly sensitive in the detection of nodules with a solid nodule detection rate of 75.9%, slightly higher than that of the other sequences. Biederer et al. (37) and Bader et al. (38) proved that the VIBE sequence can accurately detect and display solid nodules and masses in the lung atelectasis, and benign bronchial diseases. The results of a study conducted by Heye et al. (39) showed that for nodules > 6 mm in diameter, the VIBE sequence detection rate was more than 80%. The detection rate in this study was slightly low and this may be related to the large proportion of patients with sub-solid nodules seen in this study.

The HASTE sequence has certain advantages in the display of invasive lesions: the scanning speed is fast and the acquisition time is short.The sequence thus can be used to evaluate lung parenchymal lesions such as pneumonia and pulmonary edema, and can be used to identify mosaic perfusion caused by small airway diseases (40, 41). Puderbach et al. (42) demonstrated that in the case of cystic fibrosis, there was a high correlation between CT and HASTE MRI findings.

The results of a study conducted by Schroeder et al. (15) showed that the HASTE sequence was highly sensitive in detecting pulmonary nodules: for nodules with diameters of 3–5 mm, 6–10 mm, and > 10 mm, the detection rate could reach 86.3%, 95.7%, and 100% respectively. In this study, the detection rate of the T2-HASTE sequence for GGNs and PSNs was higher than that of solid nodules but overall, the detection rate was not high. This may be related to a number of factors: acquisition was greatly affected by respiration, some patients also had underlying lung diseases such as emphysema and pulmonary fibrosis to varying degrees, and cooperation during breath holding was not optimal. In addition, the acquisition was affected by the non-uniformity of the magnetic field, and this can cause blurring of the image.

The BLADE sequence is the propeller sequence: in the process of data acquisition, the combination of parallel and radial filling is adopted. This reduces image artifacts, improves the SNR of the image, and allows this sequence to satisfactorily display diseases such as pulmonary nodules and masses, lung consolidation, airway inflammation, and mucus blockage. Bruegel et al. (43) found that the solid nodule detection rate of the T2-TSE sequence for nodules with a diameter of 5–10 mm could reach 83%. The results of this study showed that the T2-BLADE sequence had a higher sensitivity and demonstrated less artifacts than the other two sequences when displaying sub-solid nodules. The detection rate of solid and sub-solid nodules with a diameter of > 8 mm was > 90%, but the relatively long scanning time is a disadvantage.

This study also revealed that, compared with the average diameter of nodules measured by CT imaging, the average diameter of nodules detected by MRI was smaller, although the difference was not statistically significant. There are a number of possible reasons for this: (1) the slice thickness of CT and MRI images is different. In order to ensure image quality, the MRI slice thickness is 3–4 mm, and there is still a certain limit in the display of small nodules, and (2) MRI images are affected by magnetic field heterogeneity and partial volume effect. For nodules containing ground glass parts, this results in the edges of the lesion being poorly displayed and therefore also affects lesion measurement.

In this study, 13 false-positive nodules (7%) were detected, and this is slightly higher than the 5% reported by Sommer et al. (4) However, in this study, the average diameter of nodules was 7.7 mm: smaller than the nodule diameter in the study above. The diameters of the false-positive nodules were < 5 mm, both were not >10 mm. The majority of false positive nodules were detected by the T2-HASTE sequence, followed by the T2-BLADE and T1-Star-VIBE sequences.

In this study, 44 of the 188 nodules were not detected, of which 20 were solid nodules, 15 were GGNs, and 9 were PSNs. Thirty-nine of these nodules were < 6 mm in diameter and the remaining 5 nodules were 6–8 mm in diameter. Undetected nodules were affected by nodule size, density, and location: the detection rate was higher for nodules in the upper lobe and the lung periphery, while nodules near the mediastinum, pericardium, and diaphragm were difficult to detect due to the influence of artifacts.

There were certain limitations to this study. First, the sample size was small: in future research, a larger sample size would be necessary to verify the diagnostic value of the sequences. Second, the baseline medical history of the patients was not evaluated, including whether underlying lung diseases, such as emphysema and pulmonary fibrosis, were present. These conditions would affect the detection of lesions to a certain extent. Third, MRI scanning was carried out only for patients with pulmonary nodules identified on CT, so the true negative results could not be evaluated, and this should be confirmed in future studies.

## Conclusion

Chest MRI can be used to detect and display pulmonary nodules. The application of multi-sequence imaging schemes, including T1-Star-VIBE, T2-HASTE, and T2-BLADE, can improve the detection rate and display performance of pulmonary nodules, especially in the case of sub-solid nodules, and MRI can be used as a supplementary investigation to CT imaging.

## Data Availability Statement

The original contributions presented in the study are included in the article/supplementary material. Further inquiries can be directed to the corresponding author.

## Ethics Statement

The studies involving human participants were reviewed and approved by ethics committee of Fourth Hospital of Hebei Medical University. The patients/participants provided their written informed consent to participate in this study. Written informed consent was obtained from the individual(s) for the publication of any potentially identifiable images or data included in this article.

## Author Contributions

Conception and design of the research: HF and GS. Acquisition of data: HL. Analysis and interpretation of the data: NZ and LW. Statistical analysis: NZ. Writing of the manuscript: HF. Critical revision of the manuscript for intellectual content: QX. All authors read and approved the final draft.

## Conflict of Interest

The authors declare that the research was conducted in the absence of any commercial or financial relationships that could be construed as a potential conflict of interest.

## Publisher’s Note

All claims expressed in this article are solely those of the authors and do not necessarily represent those of their affiliated organizations, or those of the publisher, the editors and the reviewers. Any product that may be evaluated in this article, or claim that may be made by its manufacturer, is not guaranteed or endorsed by the publisher.
